# The Clothing of Soldiers

**Published:** 1858-04

**Authors:** 


### THE CLOTHING OF SOLDIERS.

In the *Journal de la Physiologie* for January 1858, there is an excellent paper by Dr. Coulier, Professor at the Imperial School of Military Medicine and Pharmacy, entitled "Experiments upon the Materials which are used for Military Garments." The materials experimented on were as follow:

A. Cotton for shirting, coming from the firm of Waddington. Price, 0 fr. 963 c. the *mètre.*

B. Cotton for lining tunics. Waddington. Price, 0 fr. 963 c.

C. Unbleached linen for lining vests and cloaks. Price 1 fr. 75 c.

D. Dark blue cloth for soldiers' tunics and vests, 19 ains. Moules & Co. Price, 8 fr. 95 c.

E. Madder cloth for soldiers, 19 ains. Cesar Vinas. Price 8 fr. 43 c.

F. Cloth for cloaks (bluish) iron grey, 19 ains. Marcel & Renault, of Louviers. Price, 7 fr. 75 c.

H. Madder cloth for non-commissioned officers, 23 ains. Lataille Duchan, of Chateauroux. Price, 10 fr. 86 c.

K. Dark blue cloth for non-commissioned officers, 23 ains. Lataille Duchan, of Chateauroux. Price 11 fr. 40 c.

The points specially discussed by Dr. Coulier are:—

1. Materials considered as protective agents against cold.
2. Materials considered in regard to their absorbent power.
3. Clothing considered as protective agents against heat.

Dr. Coulier draws the following conclusions:

I think I have drawn from my researches the following conclusions: 1st, the colour of the clothing is without a sensible influence upon the loss of heat; 2ndly, every fabric is susceptible of absorbing, in the latent state, a certain quantity of hygrometric water,—this quantity, very considerable in wool, is less in canvas, and especially in cotton; 3rdly, this absorption takes place without immediate loss of heat to the human body; 4thly, the colour of the fabrics has a great influence upon the absorption of the solar heat; and it is sufficient, whatever else may be the nature of the clothing, to suitably modify the exterior surface in order to obtain the benefits which white materials offer during exposure to the burning heat of the sun.

